# Development of a Learning-Oriented Computer Assisted Instruction Designed to Improve Skills in the Clinical Assessment of the Nutritional Status: A Pilot Evaluation

**DOI:** 10.1371/journal.pone.0126345

**Published:** 2015-05-15

**Authors:** Laura García de Diego, Marta Cuervo, J. Alfredo Martínez

**Affiliations:** 1 Department of Food Science and Physiology, University of Navarra, Pamplona, Spain; 2 CIBERobn, Pathophysiology of Obesity and Nutrition, Institute of Health Carlos III, ISCIII, Madrid, Spain; University of Groningen, University Medical Center Groningen, NETHERLANDS

## Abstract

Computer assisted instruction (CAI) is an effective tool for evaluating and training students and professionals. In this article we will present a learning-oriented CAI, which has been developed for students and health professionals to acquire and retain new knowledge through the practice. A two-phase pilot evaluation was conducted, involving 8 nutrition experts and 30 postgraduate students, respectively. In each training session, the software developed guides users in the integral evaluation of a patient’s nutritional status and helps them to implement actions. The program includes into the format clinical tools, which can be used to recognize possible patient’s needs, to improve the clinical reasoning and to develop professional skills. Among them are assessment questionnaires and evaluation criteria, cardiovascular risk charts, clinical guidelines and photographs of various diseases. This CAI is a complete software package easy to use and versatile, aimed at clinical specialists, medical staff, scientists, educators and clinical students, which can be used as a learning tool. This application constitutes an advanced method for students and health professionals to accomplish nutritional assessments combining theoretical and empirical issues, which can be implemented in their academic curriculum.

## Introduction

The clinical science has advanced greatly in recent years, which has triggered the design of screening tools aimed at facilitating the assessment of a patients’ health status [1]. With the development of the medicine, the number of guidelines available and counseling methods, that have been developed to assist clinicians in the nutrition care process, have increased [2]. This breakthrough in medical screening tools has led to the creation of newer assessment software and the automation of very large collections of multiversion clinical guidelines [3], which has facilitated the health professionals’ work and the optimization of hospital activities [4]. Moreover, many clinical protocols have been integrated into IT-supported environments in order to develop innovative software able to generate a differential diagnoses based on clinical data [5]. However, only a small proportion of these clinical programs are available for students to improve their skills in nutritional assessment. Therefore, the healthcare software would provide a unique opportunity for the students to enhance learning during their graduate studies.

A significant proportion of learning occurs through interactive computing technologies, which has led the development of online e-learning systems, web-based learning modules or simulation programs [6–8]. In addition, the Internet Technology is widely used in continuing professional development for qualified specialists, in promoting knowledge exchange across the community and in improving the health education [9–11], being able to be considered a potential tool to evolve educational methods.

The incorporation of computer assisted instruction (CAI) in the student’s curricula reduces the instructor’s teaching time, increasing the time available to monitor individual students´ performance, improving the student’s attitude toward the course [12]. Moreover, the computer-based modules offer significant advantages in the curriculum development of the students compared to traditional teaching modalities, because provide opportunities for asynchronous learning, and allow learners access to multiple resources to further explore a topic [13]. These programs have the potential to bridge the gap between theory and practice, increasing the opportunity for students to develop problem-solving skills by allowing them to deal with simulated situations before they have to face real clinical experiences [14].

An appropriate clinical evaluation of patient’s nutritional status is accomplished when there is a correct integration between nutritional data, patient’s information and physician’s knowledge. In recent years, many of the nutritional software have been based on food consumption data collection [15], but few have helped the specialist in clinical reasoning. By the simulation, it is possible to execute this integration, and many of these applications are used as pedagogical tools for the health professionals´ education [16]. Students and nutrition specialists can learn to recognize abnormal nutritional states, to perform evaluations and to prescribe appropriate treatments to the patient by the employment of these software [17]. Moreover, the computerized clinical decision support systems help health care providers to avoid errors and, substantially, to improve clinical practice and efficiency in health care [18].

The aim of this project was to develop a Computer Aided Instruction (CAI) for the learning of nutritional assessment, featuring a multidisciplinary perspective. This CAI is part of UNyDIET (nutrition software) [19], and it consists of a complete software package that submitted instructional material related to the diagnosis of nutritional issues. Using this CAI, students can perform simulations of clinical cases of patients with diseases associated to the nutritional status. The focus was to create a software that integrates nutritional data, patient’s information and current tools of work that help the professional in the clinical reasoning based on data from previous nutritional, clinical and epidemiological studies.

## Methods

### Design

The application has been designed using the programming language Java Swing, which is a cross-platform framework. We chose this programming language because it can be used with any operating system that has a Java virtual machine (JRE 6), for example, any of the versions of Windows (95, 98, 2000, xp, etc.), Mac OS X or any of the different Linux distributions. The database is SQLite and uses some external libraries such as JfreeChart for plotting graphs. This program requires 256 MB of memory on the hard disk and a screen resolution of 1024x768px.

### The Computer Assisted Instruction

UNyDIET is a nutrition software which consists of ten modules: Patient, Anthropometry, Medical History, Biochemistry, Diet History, Diagnoses, Quality of life, Fitness, Energy Expenditure and Diet, where specifically the modules Medical History, Diagnoses and Quality of life comprise the CAI and they can be used to perform simulations of clinical cases.

UNyDIET was developed in order to obtain a learning tool to accomplish nutritional assessments, which also could be used as a working instrument in nutrition intervention programs [19]. A CAI was included in this software with the intention that students and health professionals knew and applied tools which can be used to assess patient nutritional status or/and the level of nutritional risk.

**Fig 1:**
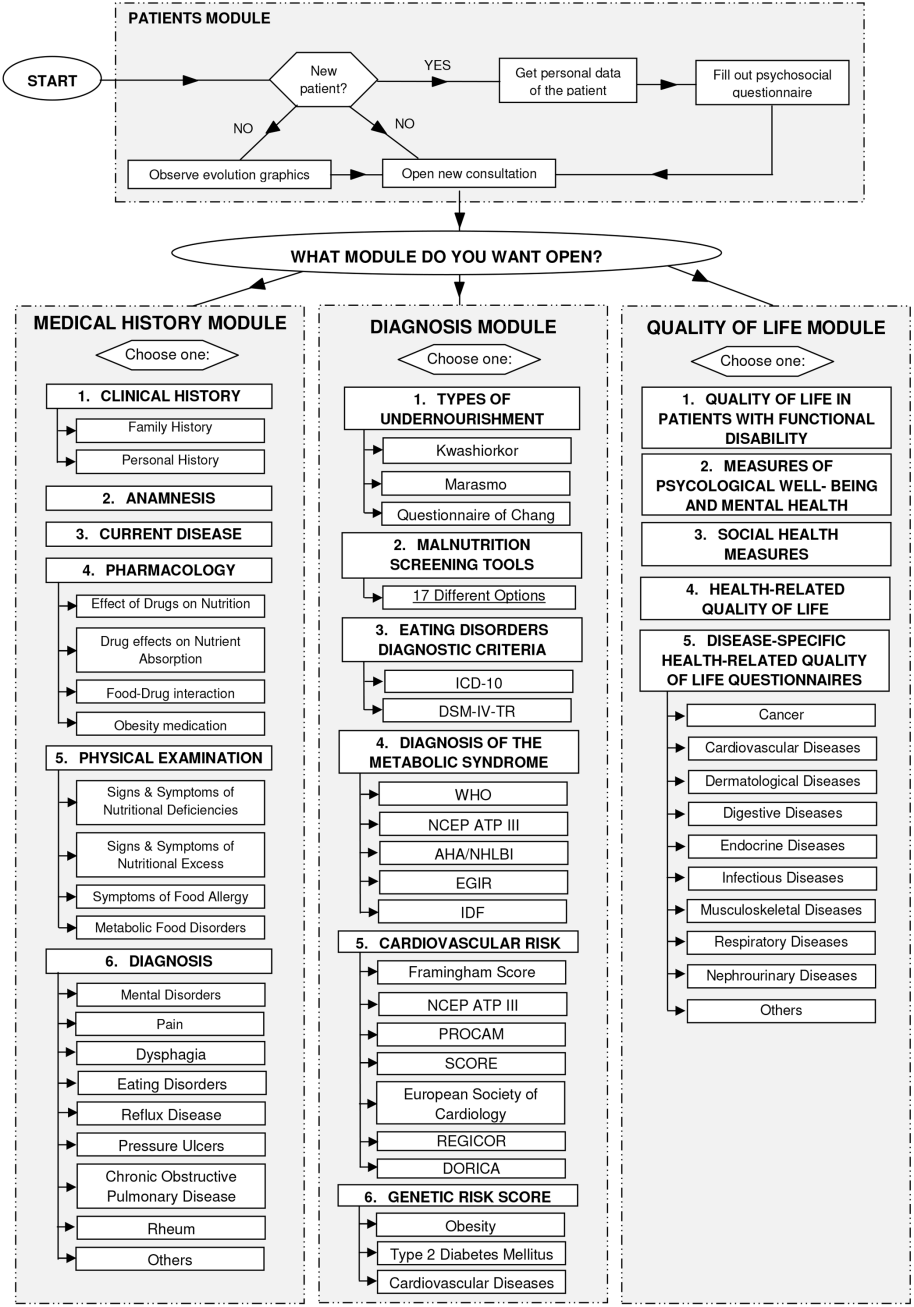
The information flow of the Computer Assisted Instruction.

The CAI was implemented in such a way that when the user enters one of these three modules, it displays a drop down menu with different hierarchical levels. The information flow of the CAI is summarized in Fig 1. According this scheme, the first step to accomplish a health status assessment is to enter in Patients module in order to discharge the file from a patient or open a file to a new patient. The next step is to enter one of the navigation buttons that will appear at the top and the bottom of the screen. Each button represents a different category of the clinical assessment of nutritional status.

**Fig 2:**
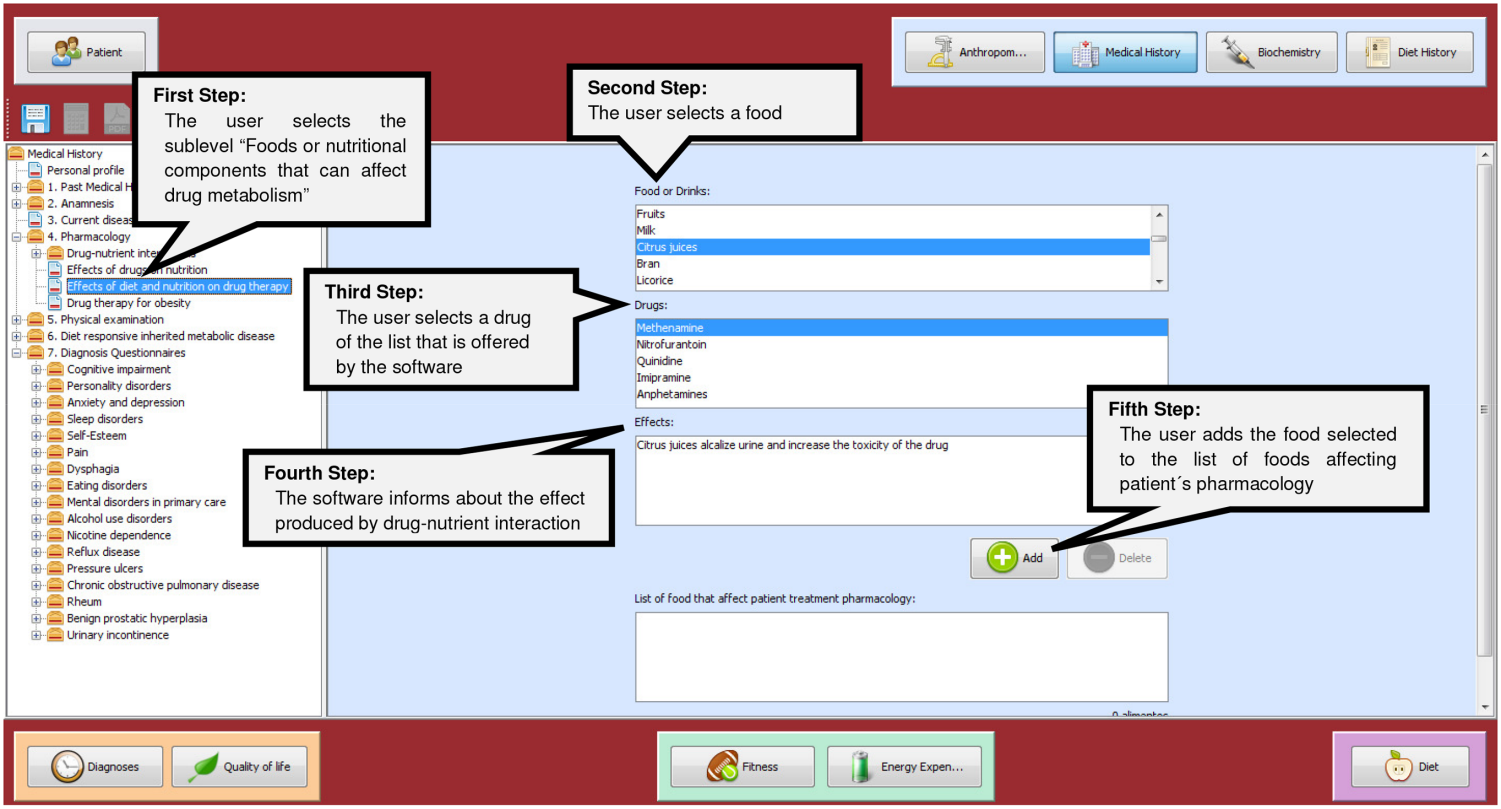
A screenshot of a section of the module of Medical History.

Inside each module a series of screen formats were developed, in which the user must type the requested data where are also provided photographs and information related to the module, an example is shown in Fig 2. An algorithm has been implemented so that as the user fills in the blanks that appear on the screens, the learning material is shown. This material, which will help the user to identify pathologies, is based on validated clinical assessment tools and guidelines. Therefore, the CAI database encompasses validated questionnaires, clinical guidelines and disease risk calculation tools to assess the patient’s health status. These instruments of evaluation are categorized within of CAI’s different modules and they are presented in Tables 1, 2 and 3.

**Table 1:** Assessment tools of the module of Medical History.

LEVEL	SUBLEVEL	ASSESSMENT TOOL
**Clinical History**		Family History Questionnaires
		Medical History Questionnaires
**Anamnesis**		Check List for Systems Review
		Stages of puberty of Tanner [20]
**Current Disease**		Symptoms, Signs and Medication
**Pharmacology**		Patient´s Medication History
**Physical examination**		A Colour Atlas of Nutritional Disorders [21]
**Diagnosis Questionnaires**	Mental Disorders	Mini-Mental State Examination (MMSE) [22]
		Short Form of the Informant Questionnaire on cognitive decline in the elderly [23]
		Pfeiffer’s test [24]
		Personality Diagnostic Questionnaire (PDQ-4+) [25]
		Personal Health Questionnaire Depression Scale (PHQ-9) [26]
		Hospital Anxiety and Depression Scale (HADS) [27]
		Hamilton Anxiety Rating Scale (HARS) [28]
		Hamilton Depression Rating Scale (HDRS) [29]
		Oviedo Sleep Questionnaire [30]
		Epworth Sleepiness Scale (ESS) [31]
		Rosenberg Self-Esteem Scale [32]
		Patient Health Questionnaire (PHQ) [33]
	Pain	Mc Gill Pain Questionnaire (MPQ) [34]
	Dysphagia	Eating Assessment Tool-10 (EAT-10) [35]
	Eating Disorders	SCOFF Questionnaire [36]
		Eating Attitudes Test-40 (EAT-40) [37]
		Eating Attitudes Test-26 (EAT-26) [38]
		Children´s Eating Attitudes Test (ChEAT) [39]
		Bulimic Investigatory Test Edinburgh (BITE) [40]
		Bulimia Test (BULIT) [41]
		Body Shape Questionnaire (BSQ) [42]
		Body Shape Dissatisfaction Assessment Scale for Adolescents (EEICA) [43]
	Reflux Disease	Carlsson-Dent Questionnaire [44]
		Reflux Disease Questionnaire [45]
	Pressure Ulcers	The Norton Scale [46]
	Chronic Obstructive Pulmonary Disease	Clinical COPD Questionnaire (CCQ) [47]
	Rheum	Health Assessment Questionnaire (HAQ) [48]
	Others	Alcohol Use Disorder Identification Test (AUDIT) [49]
		Fagerström Test for Nicotine Dependence [50]
		International Prostate Symptom Score (IPSS) [51]
		Questionnaire to Diagnose Urinary Incontinence [52]

**Table 2:** Assessment tools of the module of Diagnosis.

LEVEL	ASSESSMENT TOOL
**Types of Undernourishment**	SENPE-SEDOM document on coding of hospital hyponutrition [53]
	Questionnaire of Chang [54]
**Malnutrition Screening Tools**	Prognostic Nutritional Index (PNI) [55]
	Maastricht Index [56]
	Prognostic Inflammatory and Nutritional Index (PINI) [57]
	Instant Nutritional Assessment (INA) [58]
	Gassull Classification [59]
	Nutritional Risk Index (NRI) [60]
	Geriatric Nutritional Risk Index (GNRI) [61]
	Malnutrition Risk Scale (SCALES) [62]
	Nutritional Risk Assessment Scale (NuRAS) [63]
	Malnutrition Screening Tool (MST) [64]
	Subjective Global Assessment (SGA) [65]
	Nutrition Screening Initiative (NSI)
	•DETERMINE [66]
	•NSI Level I [67]
	•NSI Level II [67]
	Nutritional Risk Screening (NRS-2000) [68]
	Malnutrition Universal Screening Tool (MUST) [69]
	Mini Nutritional Assessment (MNA) [70]
	Mini Nutritional Assessment Short Form (MNA-SF) [71]
	Screening Tool to Identify the Nutritionally at-Risk Pregnancy [72]
**Eating Disorder Diagnostic**	Diagnostic Criteria from ICD-10 [73]
**Criteria**	Diagnostic Criteria from DSM IV-TR [74]
	Defined by World Health Organization (WHO) [75]
**Diagnostic Criteria for Identification of the Metabolic Syndrome**	Defined by the European Group for the Study of Insulin Resistance (EGIR) [76]
	Defined by NCEP ATP III [77]
	Defined by AHA/NHBI [78]
	Defined by the International Diabetes Federation (IDF) [79]
**Cardiovascular Risk**	Framingham Risk Score [80, 81]
	Coronary Risk Charts of the European Society of Cardiology [82]
	NCEP ATP III 10-year Risk Calculator [77]
	Simple Scoring Scheme for Calculating the Risk of Acute Coronary Events based on the Prospective Cardiovascular Münster (PROCAM) Study [83]
	The SCORE project [84]
	Calculation of Cardiovascular Risk on the Framingham Scale Calibrated by the REGICOR Study [85]
	Tables of Coronary Risk Evaluation adapted to the DORICA study [86]
**Genetic Risk Score**	Obesity [87, 88]
	Type II Diabetes Mellitus [89, 90]
	Cardiovascular Diseases:
	•Coronary Infarct [91]
	•Early-Onset myocardial Infarction [92]
	•Coronary Heart Disease [93, 94]
	•Coronary Artery Disease [95]
	•Coronary Artery Calcium [96]
	•Low LDL or High HDL [97]
	•Increased Level of Lp (a) Lipoprotein [98]

**Table 3:** Assessment tools of the module of Quality of Life.

LEVEL	SUBLEVEL	ASSESSMENT TOOL
**Quality of Life in Patients with**		The Barthel Index [99]
**Functional Disability**		Instrumental Activities of Daily Living Scale (IADL) [100]
		Duke Activity Status Index (DASI) [101]
**Measures of Psychological Well-Being**		Psychological General Well-Being Index (PGWB) [102]
**and Mental Health**		The Satisfaction with Life Scale [103]
		Quality of Life in Depression Scale (QLDS) [104]
		Ryff´s Scales of Psychological Well-being (SPWB) [105]
**Social Health Measures**		The family APGAR Questionnaire [106]
		Duke-UNC Functional Social Support Questionnaire (FSSQ) [107]
**Health-Related Quality of Life**		The Nottingham Health Profile [108]
		EuroQol Questionnaire (EQ-5D) [109]
		Short-Form Health Survey (SF)
		•12-Item Short Form Health Survey (SF-12) [110]
		•36-Item Short Form Health Survey (SF-36) [111]
		Quality of Life Questionnaire (CCV) [112]
		Child Health and Illness Profile (CHIP)
		•Child Report Form of the CHIP-Child Edition (CHIP-CRF) [113]
		•Parent Report Form of the CHIP-Child Edition (CHIP-PRF) [114]
		CHIP-Adolescent Edition (CHIP-AE) [115]
**Disease-Specific**	Cancer	Rotterdam Symptom Checklist [116]
**Health-Related**	Cardiovascular	Arterial Hypertension Quality of Life Questionnaire (CHAL) [117]
**Quality of Life Questionnaires**	Diseases	Short Form of the Hypertension Quality of Life Questionnaire (MINICHAL) [118]
	Dermatological Diseases	SKINDEX Questionnaire, 29 items version (SKINDEX-29) [119]
	Digestive Diseases	Inflammatory Bowel Disease Questionnaire (IBDQ)
		•32-item version of IBDQ (IBDQ-32) [120]
		•36-item version of IBDQ (IBDQ-36) [121]
		Dyspepsia-Related Health Scales (DRHS) [122]
		Chronic Liver Disease Questionnaire (CLDQ) [123]
	Endocrine Diseases	Diabetes Quality of Life Questionnaire (DQOL) [124]
		Quality of Life- Assessment of Growth Hormone Deficiency in Adults (QoL-AGHDA) [125]
	Infectious Diseases	Medical Outcomes Study HIV Health Survey (MOS-HIV) [126]
	Musculoskeletal	Fibromyalgia Impact Questionnaire (FIQ) [127]
	Diseases	Assessment of Health Related Quality of Life in Osteoporosis [128]
	Respiratory	St George´s Respiratory Questionnaire (SGRQ) [129]
	Diseases	Asthma Quality of Life Questionnaire (AQLQ) [130]
	Nephrourinary	Kidney Disease Quality of Life-Short Form (KDQOL-SF) [131]
	Diseases	King´s Health Questionnaire (KHQ) [132]
	Others	Functional Outcomes of Sleep Questionnaire (FOSQ) [133]
		Cervantes Scale [134]

**Fig 3:**
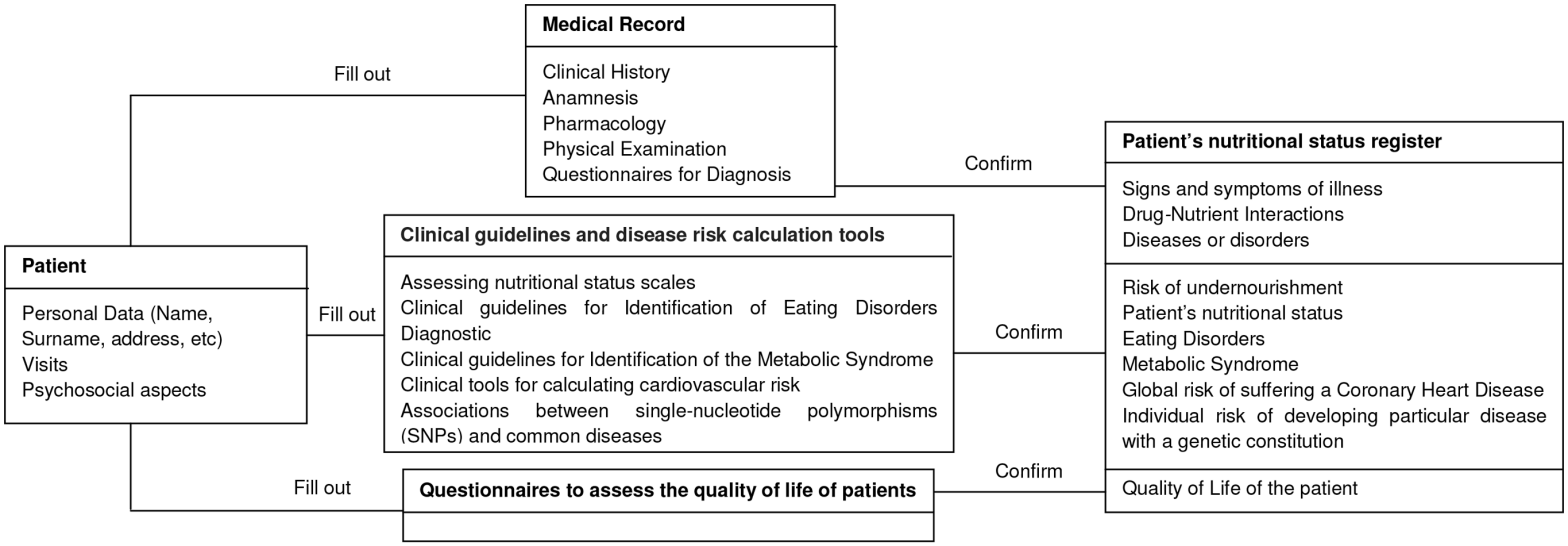
Diagram of the operational processes of the Computer Assisted Instruction.

Diagnosis tools included in the CAI are based on epidemiological studies, and they have been developed to identify diseases and to monitor a patient's progress. The CAI guides the user in performing simulations of clinical cases, reporting about: the probability that the patient has to suffer a disease, the quality of life and the risk of developing certain pathologies. Therefore, the CAI provides appraisal tools for clinical practice which facilitates the accomplishment of nutritional assessments from a multidisciplinary perspective. The Fig 3 represents the diagram of the operational processes of the CAI.

Finally, this software offers users the ability to export the information acquired to a file pdf.

In the development of this CAI the following categories were considered:

### Registration of patients

Patients module was incorporated for discharging new patients, in which the user can register the patient’s information such as name, date of birth, address and other similar information, as well as perform a psychosocial survey to the patient. For working with an existing patient, the user needs to open a new consultation to update the patient’s recorded information. An algorithm was implemented to monitor the patient’s progress which shows the graphs of key nutritional parameters.

### Medical History

The medical history or anamnesis is the factual information obtained by a physician during the exploration of a patient [135]. When the user enters the *Medical History* module, six sections are shown: Clinical history, Anamnesis, Current Disease, Pharmacology, Physical Examination and Diagnosis Questionnaires.

On Clinical History, Anamnesis and Current Disease, an algorithm was implemented that guides the user through the personal and familiar medical record. This algorithm displays a check list for systems review, in which health professionals can mark patient’s symptoms, and also lets them to enter information about the patient’s personal or familiar diseases so as to complete the medical history.

Pharmacology refers to the patient’s medication. Nutritional supplements and food that can alter medication effectiveness are also being considered in this category [136]. In this case, an algorithm was applied to show drugs categorized by the pharmacological effect that they produce (reducing or increasing appetite, causing nausea or vomiting, inhibiting the synthesis of nutrients…); and food or nutritional components that can affect drug metabolism, where the user can check a patient’s prescribed drugs and know its effect on the nutritional status, or to be informed how certain food interact with certain drugs and the metabolic effect caused by drug-nutrient interaction (Fig 2). Additionally, an algorithm was implemented to help to the user to record the food no recommended for each patient according to the prescribed pharmacological treatment.

Physical Examination. A physical exam can provide objective data that reveal nutritional deficiencies or excesses, that otherwise could not be identified. Therefore, pictures of physical signs and symptoms are shown to help the user to identify nutrient deficiency or excess, or the occurrence of a metabolic disease [21].

On Diagnosis Questionnaires, questionnaires for the diagnosis of disorders or diseases were included. Epidemiologists and clinicians have designed validated questionnaires, which are used as helpful tools to identify specific diseases [137]. Thus, the user can select several validated multiple choice questionnaires developed from epidemiological studies, which have been designed to identify various pathologies such as: cognitive impairment, personality disorders, anxiety and depression, insomnia, lack self-esteem, pain, dysphagia, eating disorders, psychiatric disorders, alcoholism, nicotine dependence, gastroesophageal reflux, pressure ulcers, pulmonary obstruction, rheumatism, benign prostatic hyperplasia and urinary incontinence (Table 1). Considering the patient’s symptoms and habits, the user can select one of the provided questionnaires of the application, which corresponds to a specific disease. After filling it in and clicking on the icon representing a calculator, the application will report on the patient’s risk of suffering the selected disease.

### Diagnoses

Screening methods, clinical practice guidelines and risk prediction equations are used to identify or calculate the risk of developing diseases such as malnutrition [138], eating disorders [139], cardiovascular disease [140] or metabolic syndrome [141].

The Diagnosis module is built on six sections (Table 2): Types of Undernourishment, Malnutrition Screening Tools, Eating Disorders Diagnostic Criteria, Diagnostic Criteria for Identification of the Metabolic Syndrome, Cardiovascular risk and Genetic Risk Score.

On Types of Undernourishment and Malnutrition Screening Tools, different assessing nutritional status scales were incorporated to determine the risk of undernourishment or just to know the patient’s nutritional status (normal, with protein-calorie deficiency or energy deficiency). Additionally, an algorithm was also coded to perform a possible diagnosis of undernourishment which was based on patient information (biochemical and anthropometric variables, dietary habits or medical history).

On Eating Disorders Diagnostic Criteria and Diagnostic Criteria for Identification of the Metabolic Syndrome, clinical guidelines in multicheck format were implemented so that the user can identify patients that suffer eating behavior disorders or metabolic syndrome, based on criteria that have been described by specialized organizations in these diseases (ICD-10, DSM-IV-TR, WHO, EGIR, NCEP-ATP III, AHA / NHLBI, IDF).

**Fig 4:**
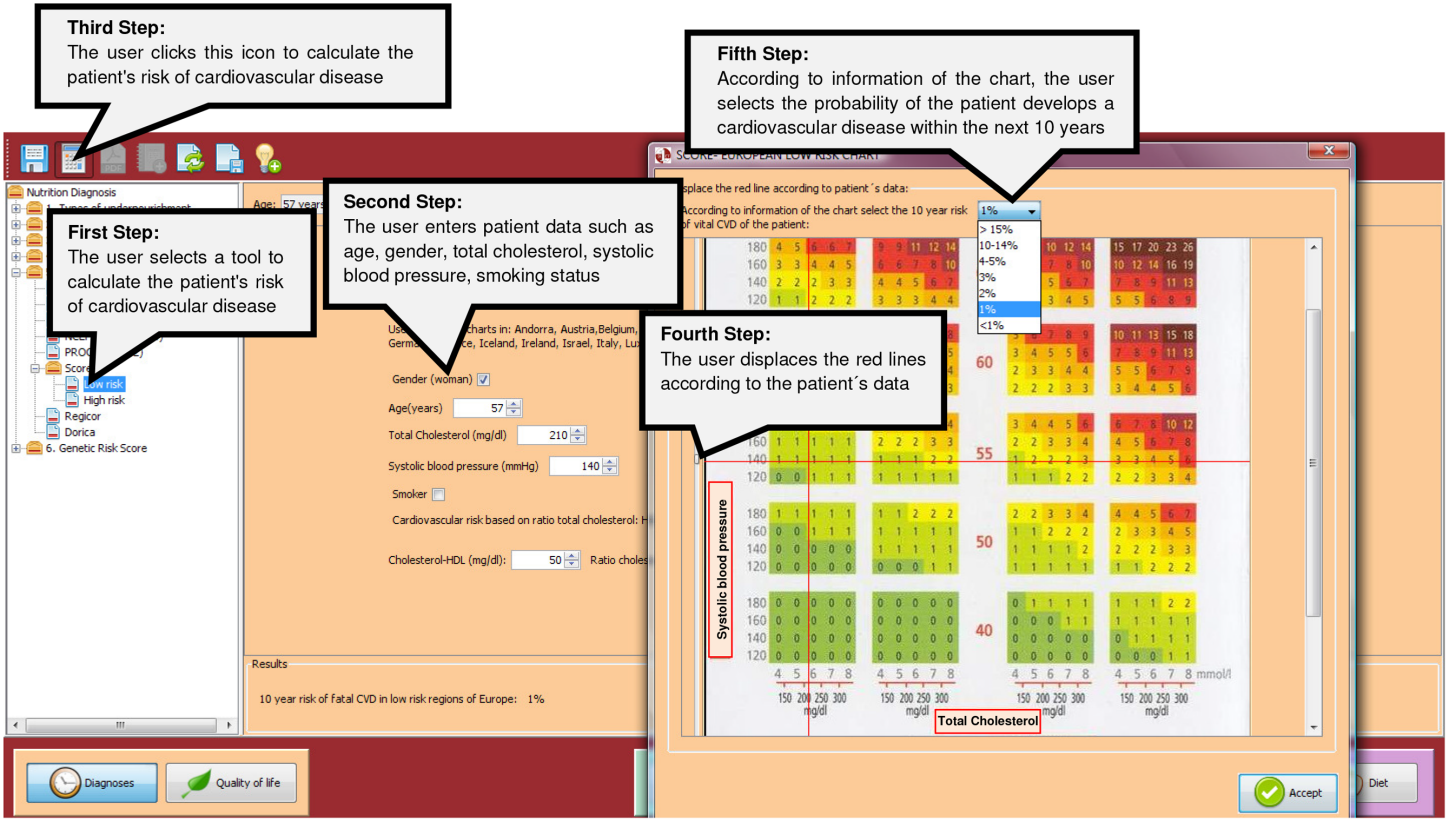
A screenshot of a risk chart of the Cardiovascular Risk section.

Tools to calculate the global risk of suffering a Coronary Heart Disease were included in Cardiovascular risk. These tools can have different formats, risk charts or calculators, depending on the epidemiological study that has been taken as a reference for their design (Framingham, European Society of Cardiology, NCEP ATP III, PROCAM, Score, Regicor, Dorica) (Fig 4). To determine the risk of coronary disease is necessary to enter patient data such as age, gender, total cholesterol, systolic blood pressure, smoking status, etc.

Genetic Risk Score. Genetic differences among individuals can affect metabolic processes and nutritional requirements. Some of these genetic differences involve alterations of individual nucleotides in the DNA sequence (SNP), which are responsible for the onset of common diseases such as obesity, type 2 diabetes and cardiovascular diseases [142]. Thus, an algorithm was implemented to enable the user to identify the individual risk of developing particular disease with a genetic constitution such as obesity, diabetes type II or cardiovascular diseases and also to know the probability that a patient is carrying a specific disease by selecting single nucleotide polymorphisms. In the concrete case of the obesity, an algorithm was incorporated to predict outcomes of patients’ weight loss based on their genetic profile.

### Quality of Life

Self- or interviewer-administered questionnaires can be used to measure cross-sectional differences in quality of life among patients at a point in time or longitudinal changes in the quality of life within patients during a period of time [143].

In the Quality of Life module, scales to measure the patient’s quality of life were incorporated, and it is constituted by the sections (Table 3): Quality of Life in Patients with Functional Disability, Measures of Psychological Well-Being and Mental Health, Social Health Measures, Health-Related Quality of Life and Disease-Specific Health-Related Quality of Life.

On Quality of Life in Patients with Functional Disability, scales which are aimed at elderly people and patients with chronic disease were implemented. The values assigned to each statement are based on the amount of actual physical assistance required by patients at the moment of fill out the questionnaire, as long as they were unable to perform an activity.

On Measures of Psychological Well-Being and Mental Health, a series of questionnaires were included so that the user could measure positive psychological health and global life satisfaction in healthy and ill patients; and the quality of life in people suffering depression.

On Social Health Measures, an algorithm was implemented so that the user could assess the role of the family in the care and the welfare of the patient.

The section Health-Related Quality of Life was composed of questionnaires, which assess self-perceived health when the patient is not suffering any disease.

On Disease-Specific Health-Related Quality of Life Questionnaires, some questionnaires were incorporated in order that the user could evaluate the patient’s quality of life related to specific diseases such as cancer, coronary, vascular, dermatological, digestive, endocrine, infectious, musculoskeletal, respiratory or nephrourinary diseases. In this section the user can perform a subjective assessment of the evolution of the disease in a patient, since improvements in the quality of life are usually accompanied by improvements in the development of the disease.

### Ethical considerations

Before starting this research, the participants were explained the purpose of the study and how the questionnaire information was going to be used. Subsequently, each potential volunteer was specifically asked if he would be willing to take part anonymously in the study. Moreover, they were also informed that they could withdraw from the study at any time. After giving them this information, the volunteers who accepted to participate signed their written consent.

This study was conducted with the approval of the Board of the Institute of Food Sciences and Nutrition of the University of Navarra, according to the guidelines laid down in the Declaration of Helsinki for anonymous surveys [144]. Later, the participants filled out the questionnaire anonymously, and their identities weren't revealed to any reader.

### Procedure

**Fig 5:**
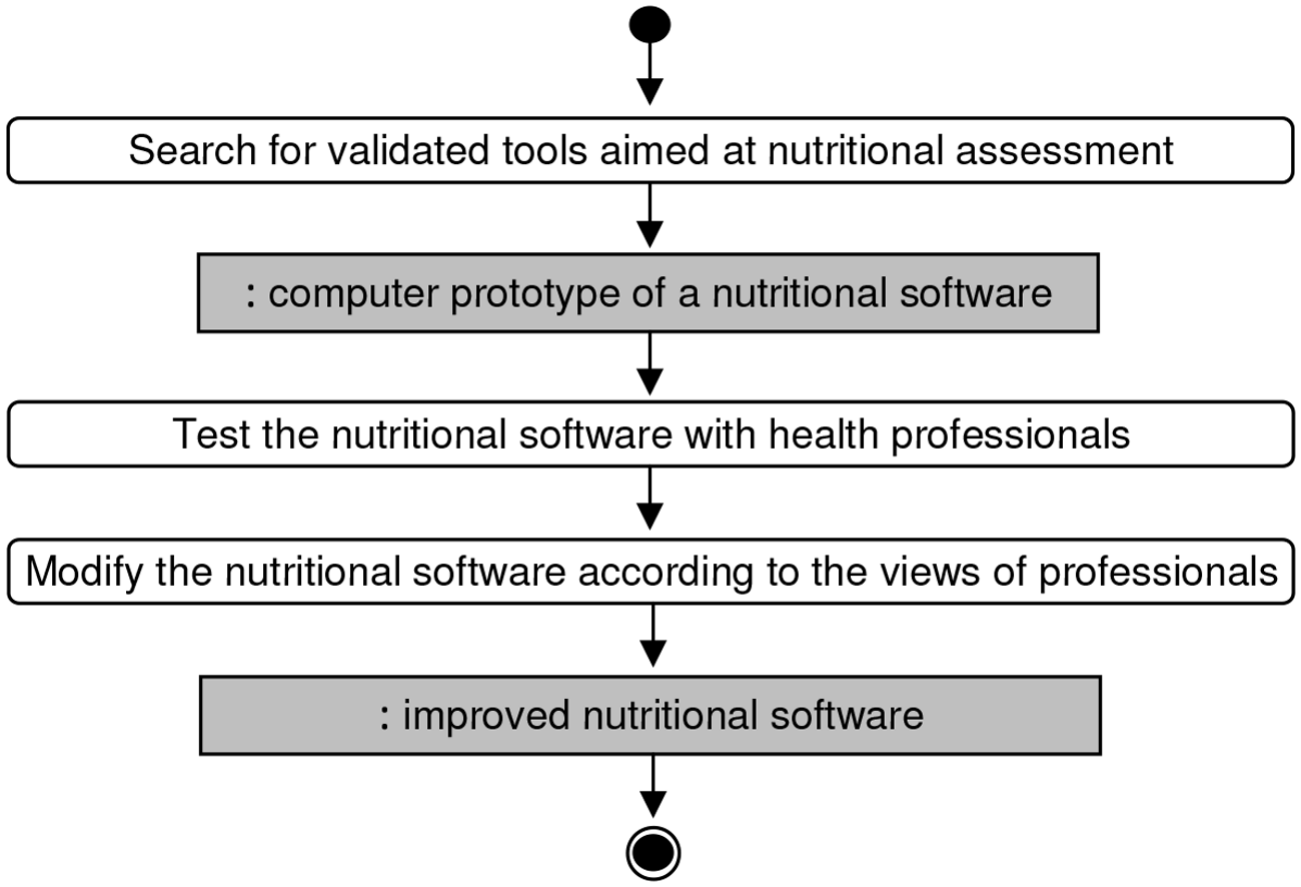
Activity Diagram for the First Phase of the assessment of the CAI.

The assessment of the CAI carried out in two phases. In the first phase a heuristic evaluation was conducted [145] (Fig 5). This pilot study involved 8 health professionals from different departments of the University of Navarra, who were recruited for their expertise in nutrition. These volunteers worked as teachers or researchers within the University. Through this evaluation we tried to find problems in user interface design, besides discussing the complexity and functionality of the software. In this phase, each evaluator had the software available during the time that they considered necessary, so that they performed a comprehensive evaluation of the CAI. The results of this evaluation were obtained by a face to face interview. Participants were encouraged to share their experiences and discuss the problems in the interface design, the complexity and functionality of the software.

**Fig 6:**
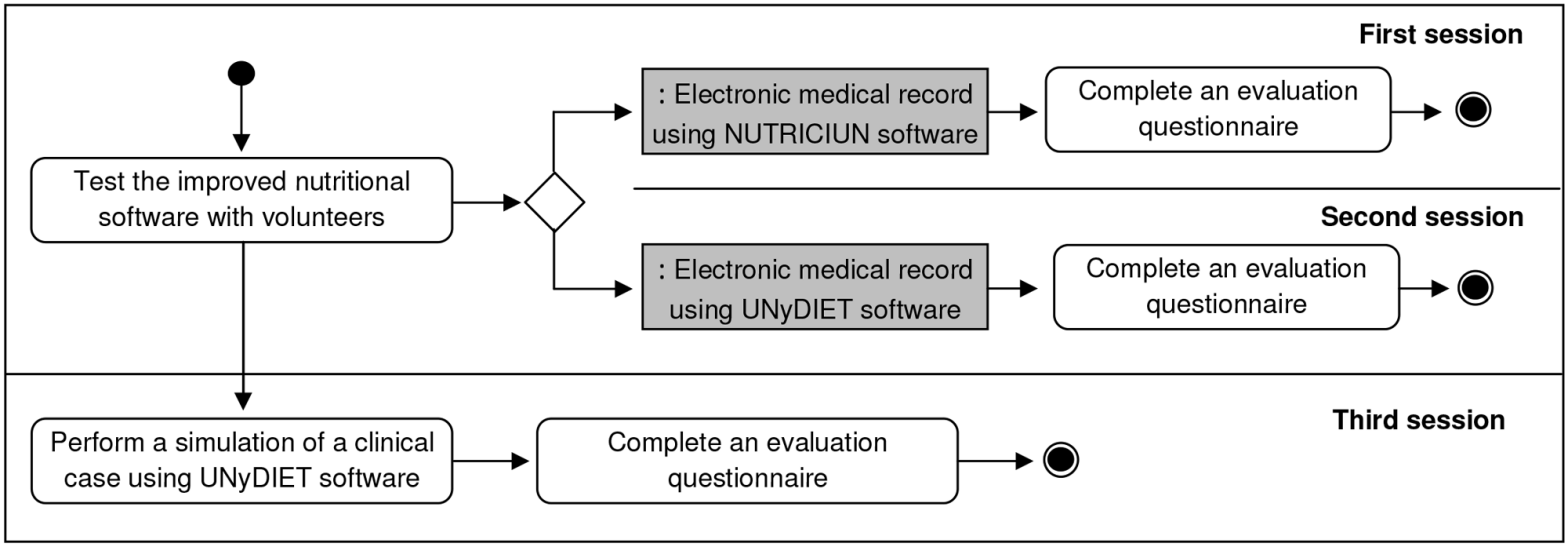
Activity Diagram for the Second Phase of the assessment of the CAI.

In the second phase of the study, 30 volunteers evaluated the usability, functionality and effectiveness of the software (Fig 6). This group was composed of 14 students of European Master's Degree in Food Science, Nutrition and Metabolism (E-MENU), 12 health sciences graduates and 4 PhD in nutritional sciences, of which 10 participants had basic, 6 good and 14 excellent nutritional knowledge. The questionnaire used and the selection of participants was conducted following the guidelines of Rubin's Handbook of Usability Testing [146]. This phase lasted about 90 minutes and each participant was working at individual computers. This part of the study consisted of three sessions:

- The object of the first session was the presentation of the nutritional software “NUTRICIUN”, which is being used currently by the University of Navarra with teaching purposes. This session was completed in 20 minutes.
- In the second session, the display of a new CAI (UNyDIET) was presented. This session was 20 minutes long.
- In the third session, the participants performed a simulation of a nutritional study adopting this new software for about 30 minutes.

At the end of each session, the volunteers had approximately 10 minutes to complete a questionnaire, which had been previously explained, to ensure that each question was clearly understood by the participants. This questionnaire was designed by team of University educators to evaluate different aspects of the CAI: functionality, usability, the application programming interface and nutritional assessment tools; and to compare this software with similar applications (i.e. NUTRICIUN).

The questionnaires were composed of semi-structured and unstructured questions, where a number of issues were covered including:

- Knowledge and attitudes towards clinical assessment tools.
- Functionality and usability of the CAI developed.
- Pros and cons of the CAI in relation to other nutritional software.
- Usefulness of the CAI to improve skills nutritional status assessment.
- Future potential directions.

### Data Collection and Data analysis

The Data Collection and the Qualitative data analysis was conducted following the guidelines of Ryan et al. on Qualitative Research [147].

Questionnaires of the second phase were composed of semi-structured and unstructured questions. In the semi-structured questions, survey respondents had to choose between yes / no / other option, justifying their answer. Subsequently, answers were coded and analysed to find trends about the usability, functionality and effectiveness of the software. With unstructured questions, we attempted that participants compared the UNyDIET and NUTRICIUN software, and opined about the pros and cons of CAI and how it could be improved. Afterwards, answers were examined to obtain emerging patterns.

At the end of the last session, the respondents were asked to assess the CAI with a number from 1 (very bad) to 10 (excellent).

## Results

In this research, we have developed a computer assisted instruction within a comprehensive nutritional assessment software (UNyDIET) in order to help students and health professionals to learn and train their confidence in handling clinical assessment instruments.

### Assessment of the software developed

The first phase of the assessment implied a face to face interview with health professionals, who appreciated the UNyDIET software in general and the CAI in particular, due to userfriendly interface and its usefulness and practicality, however they considered it was necessary to insert new clinical assessment tools and make changes on the screen format, in the programming and in writing.

The second phase of the assessment involved collecting data on questionnaires, which were filled in by master’s students, health sciences graduates and PhD in nutritional sciences. In this group, 33.3% of the participants had basic, 20% good and 46.7% excellent nutritional knowledge. Furthermore, 50% of the participants had previously used nutritional software (Nutriciun, EasyDiet, Dial, Antro, Food and Health). Some of the opinions of this group about the developed CAI were:

“It has more assessment tools, photos, genetic indexes, information on drugs than other nutritional programs”

“It allows a more complete record of the medical history and diagnostic data than other similar programs”

“It includes photographs, on the physical examination section, and assessment questionnaires which help to get a better assessment of the patient diagnoses“

“It is more complete in the area of diagnosis than other similar programs”

“There are many options in the medical history section, the software isn't only focused on dietary aspects”

“It is easy to work on a single window. The software is very complete, it considers pathological situations associated with nutrition”

“It includes more questionnaires and more photographs than other similar programs”

“It is easy to use and very visual”

“It is more complete and more intuitive than other similar programs”

From these statements, it is clear that the participants considered that the new software contained a great number of assessment tools and photographs, which can help the health professionals in carrying out a clinical history and in establishing a patient diagnosis. Furthermore, 80% of this group believed that the developed CAI was more complete than other applications of the same type.

The participants also commented the pros and cons of this CAI in relation to other nutrition software, such as Nutriciun. The pros could be classified into three categories (Table 4): easy handling, comprehensive and content, of which the last one was the most frequently cited and the most appreciated.

**Table 4:** Pros of the CAI in relation to other nutrition software.

Pros	As noted by the participants
**Easy handling**	The CAI works on a single window allowing scrolling through all modules easily. Moreover, it is very intuitive and visual
**Comprehensive**	You can make a complete medical history using this CAI, since the software isn't only focused on dietary aspects but it also allows you to record different clinical information.
**Content**	The CAI presents screening tools, graphics, photographs and diagnostic questionnaires. It also allows assessing aspects such as the quality of life or the genetic risk.

The cons of CAI were focused on two aspects of the software functionality (Table 5): the lack of a search engine and the inability to extrapolate data to statistical software.

**Table 5:** Cons of the CAI in relation to other nutrition software.

Cons	As noted by the participants
**Lack of a Search Engine**	The CAI offers the possibility to gather much information, and you can lose much time filling out all the areas if you aren’t familiarized with the software. Therefore, you can waste some time searching the fields that you want fill out.
**Inability to extrapolate data to statistical software**	If you want use this CAI for epidemiologic studies, you'll have to extrapolate clinical data to statistical software. Therefore, if the CAI hasn't an extrapolation tool, you can spend some time taking the data from the CAI to a statistical software.

**Fig 7:**
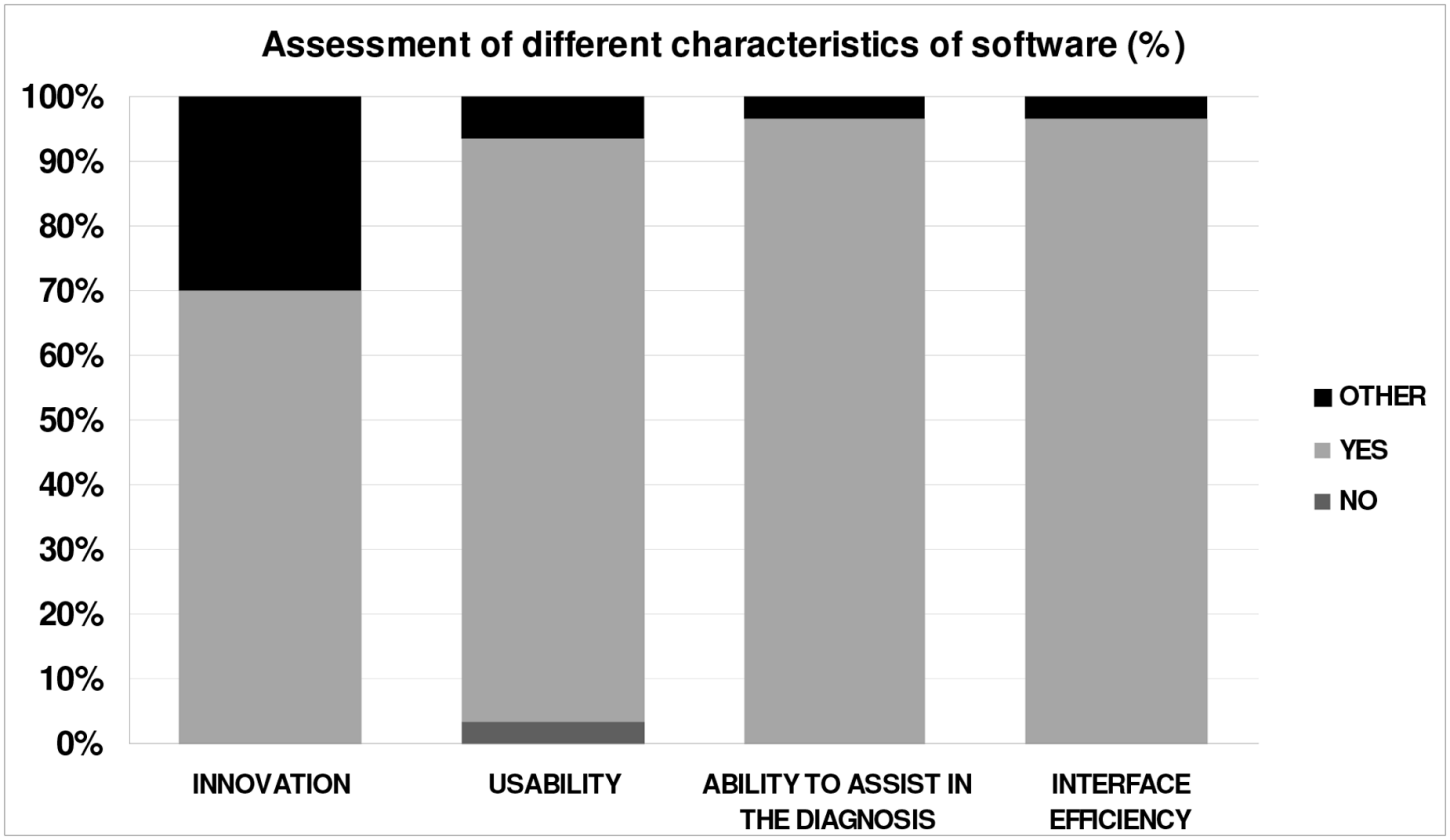
Assessment of different characteristics of software.

Participants’ surveys highlighted positively some aspects of the software: its innovation, its usability, its ability to assist in the diagnosis and the efficiency of the elements of the interface (Fig 7 and Table 6).

**Table 6:** Assessment of the different characteristics of software.

	Usuability (%)	Innovation (%)	Assist in the Diagnosis (%)	Interface Efficiency (%)
**YES**	90	70	97.7	96.7
**NO**	3.3	0	0	0
**OTHER**	6.7	30	3.3	3.3

70% of them considered the program innovative (Table 6), mainly due to its photographs, questionnaires and nutritional indexes, and the remaining 30% felt that they hadn't worked enough with other nutritional software to evaluate this characteristic.

Most of the participants felt that the developed application was useful and facilitated the carrying out of diagnoses: 97.7% of them thought the CAI helped to identify diseases and monitor a patient's progress (Table 6). 96.7% of the participants found the interface effective, mainly because it was intuitive, visual, easy to understand and friendly, and it enabled them to display the information in order (Table 6).

**Fig 8:**
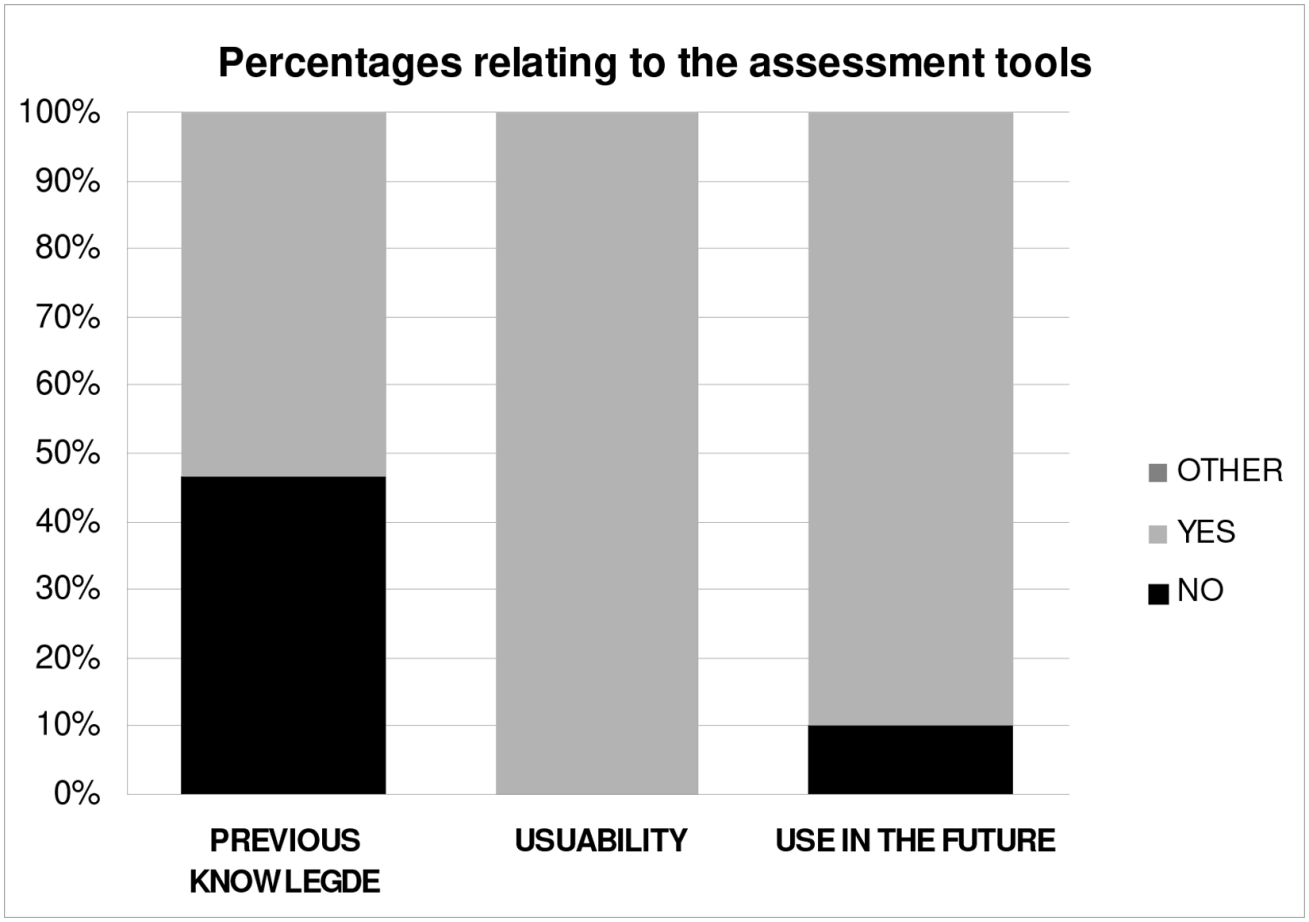
Percentages relating to the clinical assessment tools. Among respondents who would apply these tools, 33.3% would do for teaching, 37.5% in hospitals, 54.2% in dietetic consultation and 16.7% in research.

Regarding nutritional assessments tools (Fig 8 and Table 7), 46.7% of the survey respondents had never used them, but 100% considered them useful for the nutritional assessment of the patient. At the end of the second session 90% of participants stated that they would use these tools again, suggesting that performing a simulation of a clinical case with our software teaches users to manage nutritional assessment tools (Table 7). The remaining 10% of respondents wouldn´t use these tools because they don't fit to their work field.

**Table 7:** Percentages relating to the clinical assessment tools.

	Previous knowlegde	Usuability	Use in the future
**YES**	53.3	100	90
**NO**	46.7	0	10
**OTHER**	0	0	0

**Fig 9:**
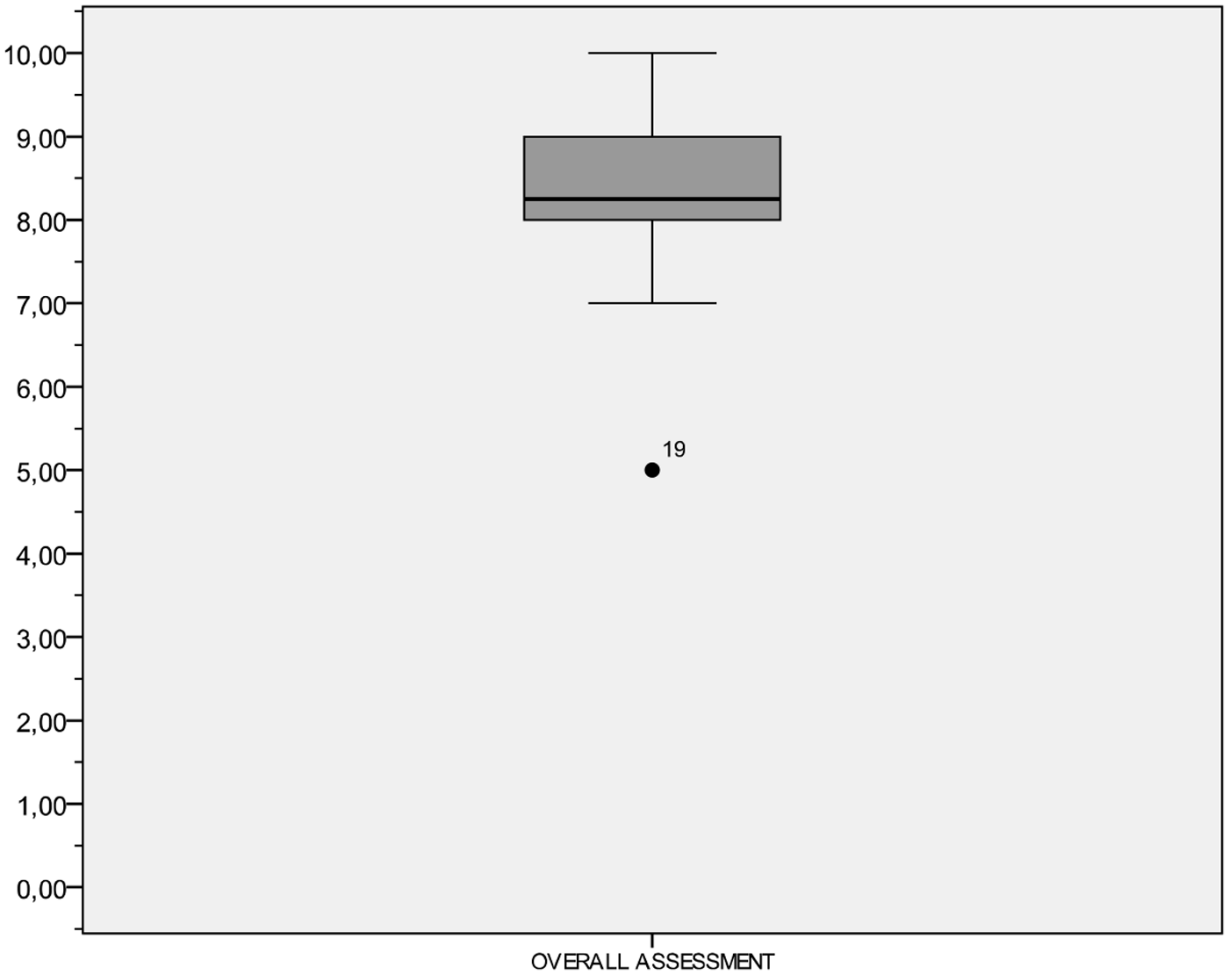
Overall assessment rating for Computer Assisted Instruction.

According to participants, the overall assessment of the CAI on a scale ranging from 1 (lowest or poorest) to 10 (highest or best) is 8.28 ± 0.99, although one of the volunteers (subject 19) gave a score of 5 (Fig 9). However, 30% of users said that the software could be improved by incorporating a search engine, and 33.3% would like to extrapolate the data to statistical software.

## Discussion

Practice, as a key component of learning and improvement of skills, is fundamental in the area of health sciences. In recent years, CAIs have been developed to improve the health status assessment skills of specialists and have been used successfully in both theory and practice settings [14].

Computer assisted medical education offers a feasible and practical method of learning [148], and for this reason students and health professionals use these applications to improve their skills in the management of people with health problems [149]. CAI modules can simulate difficult situations providing a safe and controlled learning environment to put the skills of students in practice, while avoiding unwanted effects in a real patient [150].

Clinical students sometimes aren't trained to confidently recognize, prevent and manage nutritional issues of their own patients. This is due to the fact that the skills developed within the curriculum of the junior doctors are insufficient for working within the nutritional field [151]. Therefore, the CAIs could be used to instruct healthcare professionals in the nutritional assessment, since its effectiveness for training clinical professionals has been shown on other subjects [150].

The CAI, included in the nutritional program UNYDIET, provides a step-by-step guide that leads students and health professionals during the patient's nutritional assessment, helping them to perform more accurate examinations and make better decisions in treating patients.

Most of the CAIs, used in nutritional learning, have been created to improve communication and counseling methods of the dietetic students [12] or to enhance experience of dietitians in nutrition care planning [16]. UNyDIET, unlike them, also helps clinical students in identifying diseases thanks to the inclusion of screening tools, graphics, photographs and questionnaires inside the application [19]. Therefore, the clinical tools introduced in UNyDIET not only improve clinical reasoning skills of the dietetics students referring them to the collecting of medical chart and diet history of the patient, as other CAIs [152, 153], but it also includes novel aspects related to the patient’s health status such as genetic or quality of life.

UNyDIET has integrated within a single application, the nutrition information about the patient, the risk factors or disease-related biomarkers (genetic or phenotypic) and medical knowledge [138, 142, 154]. All this will help healthcare professionals and students in disease recognition, in clinical reasoning and in prescribing treatments.

In the first phase of this research, the CAI development was given to health professionals from different clinical departments of the University of Navarra. After collecting the information of these nutrition experts, the authors improved it including new clinical assessment tools and modifying the presentation of questions and the application programming interface.

This software was also tested successfully with master’s students at University of Navarra who learned to record medical histories, to carry out a detailed physical examination, to assess the risk of disease development and to recognize the onset of diseases. After working with the CAI, they were able to recognize, more quickly, techniques and procedures required to accomplish a personalized nutritional assessment. However, the participants commented that the disadvantage that this CAI had, when compared to other dietotherapeutical sofware [155], was the lack of a module for processing statistical data.

The paucity of knowledge regarding how to integrate a CAI efficiently into clinical education causes its effectiveness to be uncertain [149], so it would be interesting that new researches were posed inside clinical settings, so the usefulness of these applications as learning tools can be established.

## Conclusions

The Computer Assisted Instruction developed offers a feasible and practical method to teach and learn about the health status assessment. Students and health professionals using this application can improve their skills in managing patients at risk of suffering health problems. The CAI included in the UNYDIET software is an electronic program, which guides the users in the data collection, analysis and interpretation, and in the identification, assessing and treatment of nutritional risks. Complete medical records and new instruments of identification of diseases are included to allow the user to know and work with new tools. Therefore, UNyDIET includes many of the categories that specialist consider important to manage a global nutritional assessment. Comparing it to other similar ones, you can see that this software helps the user in performing an integral nutritional assessment bearing in mind both genotypic and phenotypic aspects.

With the aim of improving students’ skills in clinical reasoning and in the information management, it has been decided to incorporate this CAI into the curricula of master’s students of the University of Navarra, although it would be interesting that new studies were posed to establish the usefulness of these applications as learning tools.
